# Association between dietary consumption patterns and the development of adolescent overnutrition in eastern Ethiopia: new perspectives

**DOI:** 10.3389/fnut.2023.1245477

**Published:** 2023-09-27

**Authors:** Fikerte Gedamu, Imam Dagne, Abdu Oumer

**Affiliations:** Department of Public Health, College of Medicine and Health Sciences, Dire Dawa University, Dire Dawa, Ethiopia

**Keywords:** adolescents, dietary pattern, overnutrition, principal component analysis, Ethiopia

## Abstract

**Background:**

Overnutrition among adolescents is becoming a major public health concern, with all the adverse consequences associated with unhealthy eating behaviors. Hence, clear evidence linking dietary consumption with the risk of overnutrition is crucial for targeted dietary recommendations using a robust statistical approach. This study assessed the link between dietary consumption patterns and the risks of overnutrition among adolescents in Ethiopia.

**Method:**

A community-based survey was conducted on a random sample of 510 adolescents selected using a stratified random sampling *via* proportional allocation. Dietary consumption was captured using a validated and contextualized 80-item food frequency questionnaire over the past month through a face-to-face interview. Weight and height were measured under a standard procedure. Body Mass Index for Age Z-score (BAZ) was calculated, and BAZ above +1 was considered overnutrition. The frequency measures were standardized into daily equivalents, and dietary patterns were derived using exploratory factor analysis after checking for assumptions. A bivariable and multivariable binary logistic regression model was fitted with an odds ratio and 95% confidence intervals.

**Results:**

A total of 510 participants were enrolled. Four major dietary patterns (“cereals, energy, and discretionary calory,” “fat, oil, and milk groups,” “proteins and vegetables,” and “fruits”), explaining 66.6% of the total variation, were identified. The overall prevalence of overnutrition was 29.0% (27–31%), where 22.5 and 6.5% were overweight and obese, respectively. Physical inactivity (AOR = 6.27; 95% CI: 2.75–14.3), maternal literacy (AOR = 111.3; 95% CI: 50.0–247.8), habit of snacking (AOR = 1.80; 95% CI: 0.69–4.67), skipping meals (AOR = 2.05; 955 CI: 0.84–5.04), cereals and discretionary food dietary pattern (AOR = 2.28; 95 CI: 0.94–5.55), and protein–rich and vegetable dietary pattern (AOR = 2.30; 95% CI: 0.97–5.46) were important factors associated with odds of overnutrition.

**Conclusion:**

Overnutrition is a public health concern affecting one-third of adolescents, and it is closely linked with dietary consumption patterns, eating behaviors, wealth status, literacy, and level of physical activity. Therefore, public health interventions targeting unhealthy eating and lifestyles are urgently needed to curb the increasing burden of overnutrition among adolescents and its future complications.

## 1. Introduction

Adolescents account for 16% of the global population (12 billion), of which more than 85% reside in developing countries (1). Adolescence is one of the critical life stages determining the healthy transition to adulthood, with distinct nutritional requirements and dietary habits attributable to the physiological and cognitive changes (2, 3). These huge pools of adolescents are facing an unprecedented change in food environments where processed, high-calorie, and low-nutrient foods are readily accessible and preferred (4, 5). Eating behavior, insufficient physical activity, and sedentary life during adolescence can lead to an increasing burden of overnutrition and diet-related chronic diseases (6, 7).

Overnutrition is characterized by excessive accumulation of fat as a result of excessive energy intake or decreased daily energy expenditure. The World Health Organization (WHO) defines adolescent overnutrition as body mass index (BMI) for age Z-score (BAZ) above +1, including overweight (BAZ between +1 and + 2) and obesity (BAZ above +2) (8). Due to the nutritional transition and effects of globalization, overnutrition is becoming a major public health problem, especially among adolescents (9, 10). Overnutrition among adolescents tends to persist to adulthood, and they are more likely to develop chronic diseases like diabetes mellitus, hypertension, and coronary artery disease (11, 12). Adolescents’ overweight and obesity are rising alarmingly, approaching epidemic levels in many developed countries, and have become a double burden for low- and middle-income countries (13).

The global prevalence of overweight and obesity is rising among adolescents by 14% over the past decades, affecting over 340 million adolescents, and the rate of increase is alarming in developing countries (30%) (11). Similarly, the Lancet report indicated that the number of obese girls increased from 5 million in 1979 to 50 million in 2016 and from 6 to 76 million among boys, emphasizing a huge burden. Moreover, an estimated 213 million children and adolescents are overweight (13), and the burden reaches 10.6 and 2.5% in Sub-Saharan Africa (14). In Ethiopia, the pooled prevalence of overnutrition was 11.3% (15), and different studies reported a prevalence of 9 to 26% (9, 15–18), suggesting a huge public health concern among adolescents.

Studies conducted so far have mainly focused on the effects of individual food consumption and some aspects of diet on overnutrition. However, diet could be better characterized using a robust statistical approach called “dietary pattern analysis.” It is a better approach to characterize habitual consumption and overall dietary consumption (19–23). Dietary patterns better predict the risks of overnutrition than individual food or nutrient consumption estimates. Hence, such evidence would give a more practical policy recommendation on what foods should be recommended for optimal nutrition in this context (21). Despite all its advantages, no study has been conducted in Ethiopia linking dietary patterns with adolescent overnutrition. Thus, this study was aimed at characterizing the major dietary consumption patterns and their association with overnutrition among adolescents in eastern Ethiopia.

## 2. Materials and methods

### 2.1. Study setting

This study was conducted in Dire Dawa, which is located at a latitude of 9° 35′ 35″ N, a longitude of 41° 51′ 57″ E, and 1,204 m above sea level in eastern Ethiopia. According to the Dire Dawa health bureau, the region has a total population of 535,685, and the majority of the population lives in urban areas (72%). An estimated 59,871 adolescents reside in the region, both at home in their communities and at primary and secondary schools. In urban and rural classifications, the region has nine and more than 30 kebeles (third level administrative units in Ethiopia) in urban and rural settings. The study was conducted from February to March 2022 using a primary data collection method.

### 2.2. Study design and population

A community-based cross-sectional study was conducted on a randomly selected adolescent group in Dire Dawa town to assess the relationship between various dietary patterns and the risks of overnutrition. The study findings targeted all adolescent girls in the study area, and these could indicate the burden of overnutrition and its predictors. Hence, we excluded those with vertebral deformities, spinal curvatures, and diagnosed chronic illnesses, which might affect the proper height measurement and may restrict an individual’s dietary intake.

### 2.3. Sample size determination and sampling procedure

The minimum sample size required for this study was determined for the first and second objectives and compared. A single proportion sample size estimation formula was used assuming the prevalence of overnutrition (*p* = 26.1) (17), at a 95% confidence level, 5% significance level, and 10% response rate, the final sample for the first objective became 510. For the second objective, associated factors, the minimum sample size was estimated using empirical evidence from Epi Info software for important factors affecting overnutrition among adolescents. We considered a power of 80%, a confidence level of 95%, and a significance level of 5%. Hence, taking snacking (AOR = 3.05), sweet food preferences (AOR = 2.86), and sex (AOR = 3.57), the sample sizes become 236, 296, and 210, which is smaller than the sample calculated for the first objective. Thus, we took the larger sample size of 510, calculated for the first objective, as the minimum sample size for this study.

A stratified sampling technique with a proportional allocation method was employed for this study. First, four kebeles were randomly selected using simple random sampling, and then, four blocks or villages were selected from each kebele, where the sample size is proportionally allocated relative to the number of adolescents in each village. Finally, we employed a systematic random sampling technique with a sampling fraction calculated from the total number of adolescents and the allocated sample size. With the assistance of community-based health extension workers, adolescents were tracked in the selected households.

The proportional allocation formula $ni=\left(\frac{n}{N}\right)\times Ni$ was used to allocate samples for each kebele and block, where, “*n*” is the total sample size calculated, “N” is the total adolescent population (target population), “*N*_*i*”_ is the total number of adolescents in a particular stratum, and “*n_i_*” the sample size for each stratum.

### 2.4. Variables of the study

The dependent variable was overnutrition, which is a composite indicator calculated from BAZ scores from weight and height mesurements at appropriate cutoff points. While the major dietary patterns identified from the dietary consumption captured through food frequency (FFQ) were the major independent variables of this research, socio demographic variables (age, sex, socioeconomic status, education, occupation, family size), eating behaviors (skipping breakfast, cereal products, legumes, fruit and vegetables, milk and milk products, beverages, snacks, sweet foods), sedentary lifestyles (watching television, using the computer, and playing video games), and level of physical activity (total physical activity cumulated using work, transport, and leisure time–related physical activity) were also assessed as confounding variables in assessing the association between major dietary consumption patterns with the risks of overnutrition among adolescents.

### 2.5. Operational definitions of variables

In this study, “skipping meals” was defined as the omission or lack of consumption of one or more of the traditional main meals (breakfast, lunch, or dinner) throughout the day (21). In addition, low consumption of fruits and vegetables occurs when the daily consumption of fruit and vegetables is below 400 grams (24).

Based on the standardized BAZ score, a BAZ score above +2 was classified as having obesity, while a BAZ score between + 1 SD and − 2 SD was classified as overweight adolescents and otherwise normal. Hence, the burden of overnutrition was quantified as the sum of overweight and obesity prevalence among adolescents (25).

On the other hand, the level of physical activity was assessed using the standard and highly valid General Physical Activity Questionnaire (GPAQ) and scored as a metabolic equivalent (MET) depending on the intensity and duration of each activity. Hence, we defined a physically active adolescent when the total physical activity MET minute per week is at least 600 and physically inactive otherwise (26). Thus, moderate exercises are defined based on low-impact aerobic exercise classes, brisk walking or hiking, recreational team sports (volleyball, soccer, etc.), while vigorous exercises include an activity that causes large increases in respiratory rate or heart rate like carrying or lifting heavy loads, digging or construction work, running or jogging, high–intensity aerobic classes, competitive full–field sports (soccer) or basketball (27).

### 2.6. Data collection procedures

Data were collected using a combined approach through face-to-face interviews using a structured and pretested questionnaire, anthropometric measurements of height and weight using a standard procedure. The questionnaire was prepared in English and the respective local languages. The tool is composed of socio-demographic characteristics, eating behavior, dietary pattern, and lifestyle. The data was collected by a trained pair of recently graduated health professionals.

More importantly, we employed a validated 80-item FFQ used in the Ethiopian context. The tool is modified and contextualized for the study setting, with the inclusion of important food items and the exclusion of unnecessary items. The semiqualitative FFQ includes relevant and specific food items from cereal products, legumes, fruit, vegetables, meat, milk and milk products, sweet foods, snacks, and other food consumption over the past 1 month, where the frequency is ranked from “never” to “everyday” from one to seven (22, 28). We gathered data on the frequency of consumption of various foods on each day of the previous month from all study participants. This method allows one to obtain qualitative data on the usual intake of food and the class of food over a long period of time (1 month). The FFQ validated for Ethiopian adults has been contextualized and pretested to capture the dietary intake of adults. A validation study showed that the contextualized tool was valid and reliable in measuring the dietary micro- and macronutrient intake of adults in Ethiopia (28). In pilot analysis, the FFQ tool was found to be highly reliable with a Cronbach’s alpha value above 0.86, indicative of a reproducible tool (29).

The 16-item GPAQ validated tool developed by WHO for physical activity surveillance was employed to assess the level of physical activity of adolescents in three domains, including activity at work, travel to and from places, recreational activities, and sedentary behavior, through face-to-face interviews. The activity level of the study participants was evaluated according to the standard WHO total physical activity calculation guide, and the level of total physical activity was categorized as physically active or inactive as detailed in the previous section (27, 30).

The height was measured using a Seca standing height measuring device by a trained and reliable anthropometric measurer to the nearest 0.1 cm. The weight was measured using a calibrated adult electronic scale, taking two consecutive measurements, and the average weight was recorded to the nearest 0.1 kg. Hence, the BMI was calculated by dividing the weight in kilogram by the height in meters squared. Based on this, the BMI is transformed into a standardized BAZ to rank the nutritional status of adolescents.

### 2.7. Data quality control

Data were entered into controlled data entry software (Epi Info) with validity and consistency checks. Close supervision was employed during data collection, in addition to a two-days training given to data collectors and supervisors to maintain the quality of the data. Further training of data collectors was done on proper anthropometric measurements using a standard method. Moreover, pretesting was conducted on 10 samples to evaluate the tool and assess the reliability and validity of the data collector. The technical error measurement (TEM) was calculated for each data collector, and those with a TEM above 1.5 and 2% for intra- and inter-observer measurements were excluded from collecting the data (31).

### 2.8. Data processing and analysis

Data were entered and coded using EPI–info version 7.2.1.0 for data exploration and cleaning. The cleaned data was exported to SPSS version 21 for data transformation and statistical analysis. The data were presented using frequency, percent, mean, standard deviations, statistical tables, and graphs. The data were checked for outliers, distribution skewness, and collinearity.

The age, sex, height, edema, and weight of the adolescents were further exported to WHO Anthroplus version 1.0.4 to calculate the BAZ score, where they were further exported and merged with the primary data. The BAZ score was recoded as overnourished (overweight and obesity), otherwise normal as per the standard cutoff point (25).

The FFQ was standardized and converted into a daily frequency equivalent based on previous literature. The monthly frequency of consumption was scored as 0, 0.1, 0.25, 0.571, 1, and 2-times per day (32, 33). We conducted an exploratory factor analysis (EFA) using the principal component approach using the varimax rotation. All relevant assumptions for the EFA—correlation, adequate sample, higher factor loading and absence of complex structure, were checked, and items that did not fulfill the criteria were excluded sequentially (34, 35). A Kaiser-Meyer-Olkin sample value >0.5 was considered adequate, and Bartlett’s *p*-value was below 0.05. A minimum load value above 0.3 was considered adequate for the set of variables (36, 37). Hence, we used the EFA for two purposes: to derive a ranked wealth index and to identify the major dietary patterns from the FFQ (19, 20). The wealth index was ranked into three categories: low, middle, and high.

Adolescent physical activity level was assessed in three comprehensive sets of domains, including work (moderate and vigorous), transport, and leisure time-related physical activity (moderate and vigorous). Based on the GPAQ tool, each level of physical activity was transformed into MET in minutes per week, and then the sum of sub-activities’ MET minutes per week was calculated. Then, we multiplied the number of days involved in a certain level of physical activity per week by the daily duration (in minutes) and the metabolic equivalents considering the physical activities in minutes done in a week (4.0 METS for moderate, 8.0 for vigorous and 3.0 for transport related walking or cycling) (27, 38). Hence, we calculated the total MET minutes/week and classified them to determine the physical activity level.

Bivariable and multivariable binary logistic regression were conducted to assess the associations between dependent and several independent variables. A multivariable logistic regression analysis was employed to identify factors associated with overnutrition after controlling for potential confounding variables. A crude and adjusted odds ratio with a 95% confidence interval was computed to assess the level of association between ranked dietary patterns and the risks of overnutrition. A variance inflation factor (VIF > 10) and an inflated standard error (SE > 2) were considered to assess the possibility of multicollinearity between independent variables (39). Important predictors of overnutrition found in previous studies and associations with a value of p below 0.2 in the bivariable analysis were included in the multivariate logistic regression model. Hence, we adjusted for potential confounding variables (factors) where model fitness was evaluated using Hosmer and Lemeshow’s test (value of p above 0.05) and the Omnibus test (*p*-value below 0.05) (40, 41). We also checked for an interaction or effect modification between the independent variables using a *p*-value (39). Statistical significance was declared for associations with a value of p below 0.05.

### 2.9. Ethical considerations

This study was reviewed and approved by the Dire Dawa University Institutional Research Ethical Review Board (DDU/IRB/0012/13). A letter of cooperation was taken to the respective offices before data collection. We gave a detailed and translated information sheet, and assent was obtained from the parents or guardians of adolescents aged below 18 years, while informed written consent was obtained from them directly for those aged at least 18 years. Participation in the study was on a voluntary basis, and no enforcement was implied. We conducted the interview in a secure place, and the collected data were not shared with a third party to assure confidentiality.

## 3. Results

### 3.1. Sociodemographic characteristics of participants

Of the total sample of 510 respondents, 48% were male. In addition, the majority, 208 (40.8%) and 326 (63.9%), were aged 10 to 13 years and were Muslims. Regarding maternal education, about 12.0, 16.5, 42.0, and 29.6% of them had no formal education, primary education, secondary education, or had at least a college education, respectively. Concerning occupational status, about 30.2% were housewives. About 200 (39.2%) and 161 (31.6%) of adolescents were from low and medium socioeconomic class families, respectively. Moreover, more than half (53.9%) of adolescents live in families where there are 3–5 family members (Table 1).

**Table 1:** Socio–demographic characteristics of community dwelling adolesccents in eastern Ethiopia, 2022.

Variable		Frequency	Percentage
Sex	Male	245	48%
	Female	265	52%
Age	10–13	208	40.8%
	14–16	184	36.1%
	17–19	118	23.1%
Religion	Orthodox	129	25.3%
	Muslim	326	63.9%
	Catholic	4	0.8%
	Protestant	46	9.0%
	Jewish, and adventist	5	1.0%
Mother education	No formal education	62	12.0%
	Primary education	64	16.5%
	Secondary education	214	42%
	College and above	151	29.6%
Father education	No formal education	77	15.1%
	Primary education	70	13.7%
	Secondary education	147	28.8%
	College and above	216	42.4%
Father occupation	Government employer	191	37.5%
	Merchant	135	26.5%
	Farmer	6	1.2%
	Daily labor	85	16.7%
	NGO employer	60	5%
	Self-employed	33	6.5%
Mother occupation	Government employer	152	29.8%
	Merchant	124	24.3%
	House wife	141	27.6%
	Daily labor	48	9.4%
	NGO employer	38	7.5%
	Self-employed	7	1.4%
Family size	3–5	275	53.9%
	6 and above	235	46.1%
Wealth index	Low	200	39.2%
	Medium	161	31.6%
	High	149	29.2%

### 3.2. Major dietary patterns of adolescents

Data collected using FFQ was used in PCA to assess the dietary patterns of adolescents, where the 7 food groups from my pyramid were used to find foods that correlate highly to describe particular dietary patterns in PCA. All assumptions were checked stepwise, and items not fulfilling the assumptions were excluded sequentially. The sampling adequacy was checked by Kaiser Mayer Olkin (KMO) of 0.5, and there was a significant correlation among items (X^2^ = 101, df = 21, value of p below 0.0001). Four major dietary patterns were identified, and these explained 67% of the total variation in the dietary consumption of adolescents. The identified dietary patterns had a factor loading above 0.5 in each food group, which indicates a significant correlation (Table 2).

**Table 2:** Summary of the major dietary patterns including the contribution of each food group to the total variance explained by the major dietary patterns among adolescent in eastern Ethiopia.

S.No.	Food groups	DP–1 (Factor 1)	DP–2 (Factor 2)	DP_3 (Factor 3)	DP–4 (Factor 4)
1.	Cereal and energy foods	0.684			
2.	Discretional and calories foods	0.723			
3.	Oil and fatty foods		0.853		
4.	Milk and milk products		0.592		
5.	Protein rich foods			0.842	
6.	Vegetables			0.591	
7.	Fruits				0.924
Total variance (66.9%)		20.8%	16.1%	15.5%	14.5%

DP refers to major dietary patterns derived from the reported individual food consumption.

Food group items with a complex structure (a higher loading for more than one factor) were excluded, and we derived four major dietary patterns with dominant food item loadings for each component. These are cereal, energy, and discretionary calorie foods; fatty and milk products; protein-rich foods and vegetables; and fruits. A factor score was generated using the Bartlett procedure, which is a robust and unbiased estimate of the true factor score. Then, the factor score was categorized into three terciles (low, medium, and high). Four dietary patterns, including “cereal, energy, and discretionary calorie foods,” “oil, fatty foods, and milk products,” “protein-rich foods, and vegetables,” and “fruits,” were identified as indicated in Table 2.

### 3.3. Prevalence of overnutrition among adolescents

In this study, a total of 115 (22.5%) and 33 (6.5%) were found to be affected by overweight and obesity, respectively. The combined prevalence of overnutrition (both overweight and obesity) was 29% (95% CI: 27–31%). Related to these, 2% of adolescents were found to be thin. When disaggregated by certain relevant factors, the burden of overnutrition was higher among females (15.6%) and among mid-adolescents aged 14–16 years (10.3%) as compared to males (13.1%) and older adolescents (7.1%), respectively. More importantly, the burden was significantly higher among private schools (22.5%) as compared to those who attend public schools (3.6%), excluding those who did not attend school during the study.

### 3.4. Factor associated with over–nutrition among adolescents

A bivariable binary logistic regression was conducted, and the association between socio-demographic, dietary/eating habits, physical activity, sedentary lifestyle, and dietary consumption pattern related factors with overnutrition among adolescents was evaluated. Hence, the state of overnutrition was significantly associated with maternal education, higher wealth status, family size, habits of snacking, skipping breakfast, being physically inactive, sedentary life, and a dietary pattern characterized by cereal and energy-rich foods at a *value of p* of less than 0.05.

Older adolescents (COR = 1.23; 95% CI: 0.75–2.01), females (COR = 1.13; 95% CI: 0.77–1.65), and those from the wealthiest families (COR = 1.58; 95% CI: 1.00–2.50) had a higher risk of overnutrition as compared to their counterparts. In addition, adolescents from literate families (COR = 94.3; 95% CI: 50.1–177.6) and smaller family sizes (COR = 1.59; 95% CI: 1.03–2.46) were significantly associated with a higher burden of overnutrition compared to adolescents from illiterate families and larger family sizes (> 5). Furthermore, snacking (COR = 2.68; 95% CI: 1.70–4.23) and skipping breakfast (COR = 2.56; 95% CI: 1.68–3.89) significantly increased the risk of overnutrition by 2.7- and 2.6-folds, respectively. Physical inactivity (MET below 600 MET minutes per week) (COR = 1.61; 95% CI: 1.08–2.39) and sedentary lifestyle (COR = 4.68; 95% CI: 2.96–7.42) were significant factors positively associated with the risks of overnutrition among adolescents (Table 3).

**Table 3:** Bivariable logistic regression analysis of factors associated with over nutrition among adolescents in eastern Ethiopia, 2022.

Factors	Options	Overnutrition		COR (95% CI)	*p–value*
		Yes	No		
Age of the respondents in years	10–13	58	150	1	
	14–16	52	132	1.02 (0.66–1.58)	0.934
	17–19	38	80	1.23(0.75–2.01)	0.411
Sex	Male	68	177	1	
	Female	80	185	1.13 (0.77–1.65)	0.545
Wealth index	Low	54	146	1	
	Medium	39	122	0.86 (0.54–1.39)	0.549
	High	55	94	1.58(1.00–2.50)	0.049^*^
Maternal education	Illiterate	20	339	1	
	Literate	128	23	94.3 (50.1–177.6)	0.0001^**^
Family size	<5	85	175	1.59 (1.03–2.46)	0.036^*^
	> =5	63	187	1	
Skipping breakfast	Yes	109	189	2.56 (1.68–3.89)	0.0001^**^
	No	39	173	1	
Snacking	Yes	119	219	2.68 (1.70–4.23)	0.0001^**^
	No	29	143	1	
Physical activity level (MET min/week)	< 600	98	199	1.61 (1.08–2.39)	0.020^*^
	≥ 600	50	163	1	
Sedentary life	< 3 h	120	173	4.68 (2.96–7.42)	0.0001^**^
	>3 h	28	189	1	
Cereal and energy foods (DP–1)	Low	58	112	1.80 (1.11–2.91)	0.017^*^
	Medium	52	118	1.53 (0.94–2.49)	0.086
	High	38	132	1	
Fatty foods and milk (DP–2)	Low	51	119	1	
	Medium	41	129	0.74 (0.46–1.20)	0.223
	High	56	114	1.15 (0.73–1.81)	0.559
Protein rich and vegetables (DP–3)	Low	51	119	1.35 (0.83–2.18)	0.223
	Medium	56	114	1.55 (0.96–2.49)	0.072
	High	41	129	1	
Fruits (DP–4)	Low	51	119	1	
	Medium	41	129	0.78 (0.48–1.27)	0.319
	High	56	114	1.50 (0.95–2.34)	0.082

1–refers to the reference category and MET indicates metabolic equivalents in minutes per week; DP–refers to the major dietary Patterns derived through exploratory factor analysis. Also, ^*^ refers to significant factors at a *p*–value below 0.05 (^*^) and 0.001 (^**^).

Regarding the association between major dietary patterns and the risks of overnutrition, low and medium percentiles of cereal and energy food consumption patterns (COR = 1.53; 95% CI: 0.94–2.49) and higher percentiles of fatty food consumption patterns (COR = 1.15; 95% CI: 0.73–1.81) were associated with a higher risk of overnutrition among adolescents as compared to those with low terciles of energy and fatty food consumption. While those with low (COR = 1.35; 95% CI: 0.83–2.18) and medium protein-rich foods and vegetable consumptions (COR = 1.55; 95% CI: 0.96–2.49) were positively associated with a higher odd of overnutrition among adolescents (Table 3).

Variables associated with overnutrition in bivariable analysis and important factors explored in previous literature were included and tested in multivariable binary logistic regression analysis. A Hosmer and Lemeshow’s goodness of fit value of 0.98 was identified, which indicates a fitted regression model for a given dataset. In addition, the effects of further addition or removal of factors on the model fitness were evaluated using an omnibus test (*value of p* < 0.0001), indicating an improved model. Variables with a significant decline in model fitness on removal were retained in the final model despite a higher *value of p* as shown in Table 4.

**Table 4:** Multivariable logistic regression analysis output indicating the association between major dietary consumption patterns and other lifestyle factors with the development of overnutrition among adolescent in eastern Ethiopia, 2022.

Factors	Options	Overnutrition		AOR (95% CI)	*P–value*
		Yes	No		
Sex	Male	68	177	1	
	Female	80	185	1.32 (0.63–2.76)	0.462
Wealth index	Low	54	146	1	
	Medium	39	122	1.50 (0.62–3.62)	0. 373
	High	55	94	1.82 (0.75–4.42)	0. 187
Maternal education	Illiterate	20	339	1	
	Literate	128	23	111.3 (50.0–247.8)	0.0001**
Family size	<5	85	175	2.00 (0.89–4.48)	0.092
	> = 5	63	187	1	
Skipping breakfast	Yes	109	189	2.05(0.84–5.04)	0.117
	No	39	173	1	
Snacking	Yes	119	219	1.80 (0.69–4.67)	0.0230*
	No	29	143	1	
Physical activity level (MET minutes/week)	< 600	98	199	6.27 (2.75–14.3)	0.0001**
	≥ 600	50	163	1	
Sedentary life	< 3 h	120	173	6.41 (2.84–14.5)	0.0001**
	>3 h	28	189	1	
Cereal and energy foods (DP–1)	Low	58	112	1.69 (0.71–4.04)	0.236
	Medium	52	118	2.28 (0.94–5.55)	0.070
	High	38	132	1	
Protein rich and vegetables (DP–3)	Low	51	119	1.06 (0.44–2.55)	0.891
	Medium	56	114	2.30 (0.97–5.46)	0.058
	High	41	129	1	

^**^ refers to associations with a *p*–value below 0.001 (^**^) and 0.05 (^*^).

Based on fitting a regression model, we found that maternal literacy, family wealth status, physical activity, sedentary lifestyle, snacking, skipping breakfast, and four major dietary patterns were found to be important factors that could potentially predict risks of overnutrition among adolescents. For instance, adolescents from the highest wealth quintile (AOR = 1.82; 95% CI: 0.75–4.42) and less extended families (AOR = 2.00; 95% CI: 0.89–4.48) had an almost twofold increased risk of overnutrition compared to their counterparts. Physical inactivity (600 MET minutes per week) was associated with a more than sixfold increase in the risk of overnutrition (AOR = 6.27; 95% CI: 2.75–14.3) when compared to those who were physically active. Adolescents from educated families (literate mothers) were statistically significantly associated with a higher occurrence of overnutrition (AOR = 111.3; 95% CI: 50.0–247.8), indicating the majority of the overnutrition cases were concentrated among adolescents from educated families. Moreover, those with a habit of snacking (AOR = 1.80; 95% CI: 0.69–4.67) and skipping breakfast (AOR = 2.05; 955 CI: 0.84–5.04) were significantly associated with a twofold increased risk of overnutrition compared to those without habits of snacking and those with a regular meal. As compared to those with higher percentiles of cereals and discretionary food consumption patterns, adolescents with low (AOR = 1.69; 95 CI: 0.71–4.04) and medium (AOR = 2.28; 95 CI: 0.94–5.55) cereal consumption had 69 and 128% higher risks of being overnourished, respectively. Adolescents with a relatively medium tercile of protein-rich and vegetable consumption (medium) have a 2.3-times higher risk of overnutrition (AOR = 2.30; 95% CI: 0.97–5.46) as compared to those with a higher percentile of protein-rich and vegetable consumption. Hence, a higher tercile of protein-rich and vegetable consumption lowered the risks of overnutrition among adolescents (Table 4).

## 4. Discussion

Overnutrition is considered a global epidemic, and the burden is increasing (1, 2). It is a major risk factor for a number of chronic diseases, including type 2 diabetes, cardiovascular disease, hypertension, dyslipidemia, and premature death (3, 4). Hence, evaluation of the role of nutritional and eating behaviors in reducing the risks of overnutrition is crucial for better dietary recommendations. Therefore, the purpose of this study was to assess the burden of overnutrition and identify the association between different dietary patterns and the risks of overnutrition among adolescents. According to our study, 22.5 and 6.5% of adolescents were victims of overweight and obesity, with a total burden of overnutrition reaching 29%. The prevalence of overweight and obesity in this study is higher than the prevalence reported by studies conducted in Addis Ababa (5), Gondar (6), Jimma (7), Bahir Dar (8), and Dire Dawa (9), which indicated 10–21% of adolescents had overnutrition in different parts of Ethiopia. As a result, overnutrition is a major public health issue that warrants more comprehensive and effective preventive strategies to halt its progression (10, 11). In addition, the hot weather conditions of the study area might have greatly affected the lifestyle, minimizing physical activities and walks with a preference for sweet foods produced in the area (12). Moreover, people residing in hot environment tends to consume fasting, fried food, and unfortunately, sweet food productions are common in the study area that may increase the risk. Partly due to weather, people may tend to consume foods in streets which are mainly unhealthy diets.

Overnutrition was found to be a major problem among adolescents from literate and wealthier families. A study among women also showed that there is a significant interaction between education and wealth for predicting obesity risks (value of *p* <0.0001). It is also indicated that the impact of wealth on the risk of obesity is higher for those with lower educational status (AOR = 1.78) than for those who are well-educated (13, 14). This might be due to the fact that adolescents from uneducated families have poor dietary behavior and are more likely to be stunted during early life (15). Hence, exposure to a highly nutritious diet with increasing wealth later may impose a severe risk of obesity. This might be linked to the role of early nutritional insults in the later development of obesity and other cardiovascular complications (16, 17). On the contrary, risk of sedentary behaviors and consumptions of ultra-processed and high energy foods are more common among the literate and the wealthier families due to access and price affordability.

Adolescents with a habit of snacking and skipping breakfast had almost a twofold increased risk of overnutrition among adolescents. In previous studies, skipping breakfast and snacking were associated with an increased risk of overnutrition (9, 18). In addition, skipping meals could increase the risk of obesity by fourfold (AOR = 3.55, *p*-value <0.05) (19, 20). A systematic review and meta–analysis from China using data from different countries confirmed that skipping breakfast is 1.44 times more likely to increase the risk of overweight or obesity than non-skipping breakfast (21). Adolescents having ≥3 snacks per day tend to consume more calories and be less physically active, with a higher total energy intake compared to those with a regular meal. Snacking, according to previous research, increases the risk of overnutrition and its associated health risks (20, 22). However, the type and size of snacks play a significant role in predicting overnutrition risks, as high-quality and low-energy snacks may supplement daily physiological requirements and diet quality (19, 23). On the other hand, we found that a higher number of adolescents reported skipping breakfast. Evidence also showed that a healthy breakfast could create satiety, reduce binge eating, and allow people to have optimal weight (24, 25). Hence, adolescents should not skip meals, as it affects school performance and health consumption, in addition to higher tendency for junk food consumption (26).

In this study, we found that lower consumption of cereals and higher consumption of discretionary food dietary patterns determine the risk of overnutrition. In addition, lower tercile of protein rich and vegetable dietary patterns were important factors associated with overnutrition among adolescents. A study from China also indicated that subjects in the highest quartiles had a higher risk of obesity (27). It is clear that wholegrain cereal consumption rich in dietary fiber has the potential to limit energy consumption and decrease glucose and cholesterol absorption (28, 29). On the other hand, foods rich in protein and vegetables could have a weight control effect by maintaining a healthy diet and optimum weight. Hence, diets rich in cereals, protein, and vegetables could have paramount importance in preventing overnutrition among adolescents (30). However, adolescents’ dietary behavior may tend to favor unhealthy dietary preferences such as discretionary, high-calorie, and processed foods (31). This would be high among those who skip meals and have snack, where they consume high energy and processed foods that predispose adolescents to higher risks of overnutrition (23, 32). This would be further aggravated by the influence of peers (33, 34).

Physical activity (PA) and a sedentary lifestyle, are among causes of overweight or obesity in teenagers. A sedentary lifestyle is quite relaxed, including sitting, lying down, etc., every day at work (working at the computer, reading, etc.) and at home (watching TV, playing games, etc.) (35, 36). There is no consensus about a single cutoff point to define PA to prevent overweight and obesity, but one of the main factors contributing to increased adiposity is lower energy expenditure caused by decreased PA (37, 38). In this study, the odds of being overweight or obesity among adolescents with higher hours of sedentary behaviors were 6 times more likely than those who did not have a sedentary life. This finding is higher than the study conducted in Jimma and Dire Dawa, which found that children and adolescents who watched TV for more than 4 h a day (7, 9) were more prone to obesity than those who watched for less than 4 h/day (39). This finding might be related to the lack of physical activity, which causes low energy expenditure and ultimately predicts the risk of obesity (40). The physical activity level of adolescents is low in urban setting below the daily minimum of 600MET per week. This could be further limited by the hoot weather condition of the study area. One study has showed that only 28% has the recommended PA level and less than 50% use walk from home to school and this worst in the urban setting (41).

Generally, dietary consumption patterns, physical activity level, snacking, skipping meals, and maternal literacy were associated with the risks of overnutrition among adolescents. Proper characterization of the diet is critical in linking dietary risk factors with overnutrition among adolescents (42, 43). Dietary consumption could be better characterized using dietary pattern analysis, which is a new nutritional epidemiological approach that can better predict nutritional outcomes than individual food and nutrient intakes (44). This study gives the first insight into how to create a link between dietary patterns and the risks of overnutrition among adolescents. Hence, the findings of this study would help in designing more practical dietary recommendations for the optimal nutrition of adolescents.

Although this study generates valid information quantifying the relationship between eating behavior and overnutrition among adolescents using a reliable method, the use of cross-sectional data might make it difficult to establish causality (45, 46). The current study is well conducted in a large adolescents’ cohort from the same geographic area, however assessing adolescents’ habits over the last month might not show their usual habits and the findings of this study may tend to have some limitations. Some respondent biases, inherent errors in anthropometric measurements, and social desirability bias could not be avoided totally. Moreover, respondents may tend to respond positively or negatively to the daily dietary consumption, which might bias the overall diet quality and be associated with overnutrition (47, 48). Hence, it is imperative to consider such limitations in interpreting the results of the current study.

## 5. Conclusions and recommendations

Overnutrition is a public health concern affecting one-third of adolescents, and it is closely linked with dietary consumption patterns, skipping breakfast, the habit of snacking, wealth status, literacy, and level of physical activity. Therefore, public health interventions to curb overnutrition among adolescents targeting unhealthy eating patterns and lifestyles are urgently needed to halt its emergence. Furthermore, parents of adolescents should promote an enabling environment for healthy dietary consumption and a better nutritional status for their children. Schools and the health bureau should strengthen access to physical activity facilities and regular physical activities through school competitions and healthy clubs.

## Data availability statement

The original contributions presented in the study are included in the article/supplementary material, further inquiries can be directed to the corresponding author.

## Ethics statement

The studies involving humans were approved by Dire Dawa Univesity Institutionl Review Board. The studies were conducted in accordance with the local legislation and institutional requirements. Written informed consent for participation in this study was provided by the participants' legal guardians/next of kin.

## Author contributions

FG participated in conceptualization, design, data curation, project administration, data analysis, writing a report, and reviewing and approving the manuscript. AO participated in conceptualization, design, validation, supervision, methodology, data acquisition, data preparation, data visualization, and formal data analysis. In addition, AO substantially contributed to writing the draft manuscript, manuscript preparation, reviewing, and submitting the manuscript. ID participated in drafting the manuscript, critically reviewing it, and editing the drafted manuscript. All authors contributed to the article and approved the submitted version.

## Figures and Tables

**Table:** 

A/COR	Adjusted/crude/Odds Ratio
BMI	Body Mass Index
BAZ	Body Mass Index for Age
CI	Confidence Interval
CVD	Cardiovascular Disease
DP	Dietary Patterns
EFA	Exploratory Factor Analysis
FFQ	Food Frequency Questionnaire
GPAQ	General Physical Activity Questionnaire
METS	Metabolic Equivalents
PCA	Principal Component Analysis
NCDs	Non communicable Diseases
SD	Standard Deviations
SE	Standard Error
TEM	Technical Error of Measurement
UNICEF	United Nations International Children Education Fund
VIF	Variance Inflation Factor
WHO	World Health Organization
